# The Relationship between Microbial Communities in Coffee Fermentation and Aroma with Metabolite Attributes of Finished Products

**DOI:** 10.3390/foods13152332

**Published:** 2024-07-24

**Authors:** Tatsaporn Todhanakasem, Ngo Van Tai, Soisuda Pornpukdeewattana, Theppanya Charoenrat, Briana M. Young, Songsak Wattanachaisaereekul

**Affiliations:** 1 School of Food Industry, King Mongkut’s Institute of Technology Ladkrabang, Bangkok 10520, Thailand; 64608035@kmitl.ac.th (N.V.T.); soisuda.po@kmitl.ac.th (S.P.); songsak.wa@kmitl.ac.th (S.W.); 2 Department of Biotechnology, Faculty of Science and Technology, Thammasat University (Rangsit Centre), Bangkok 10200, Thailand; thep@tu.ac.th; 3 Department of Medical Microbiology and Immunology, School of Medicine, University of California at Davis, One Shields Ave., Davis, CA 95616, USA; bmyoung@ucdavis.edu

**Keywords:** coffee, metagenomic, microbial communities, biochemical compounds, aroma

## Abstract

Coffee is a critical agricultural commodity and is used to produce premium beverages enjoyed by people worldwide. The microbiome of coffee beans has proven to be an essential tool that improves the flavor profile of coffee by creating aromatic flavor compounds through natural fermentation. This study investigated the natural microbial consortium during the wet process fermentation of coffee onsite in Thailand in order to identify the correlation between microbial diversity and biochemical characteristics including flavor, aroma, and metabolic attributes. Our study found 64 genera of bacteria and 59 genera of yeast/fungi present during the fermentation process. Group of microbes, mainly yeast and lactic acid bacteria, that predominated in the process were significantly correlated with preferable flavor and aroma compounds, including linalyl formate, linalool, cis-isoeugenol, trans-geraniol, and (-)-isopulegol. Some of the detected metabolites were found to be active compounds which could play a role in health.

## 1. Introduction

Coffee, beloved worldwide for its distinct taste and aroma, owes much of its flavor to the intricate microbial communities formed during fermentation. Thailand, a hub for coffee cultivation, primarily grows three varieties: Robusta, Arabica, and hybrid. With Arabica thriving in the northern provinces like Chiang Rai, Chiang Mai, and Nan and Robusta favoring the southern provinces such as Chumphon and Ranong, the region’s landscape and climate set the stage for high coffee productivity. In 2022, coffee plantations spanning approximately 387.94 km^2^ contributed over USD 57 million to the economy, producing nearly 18,689 tons of coffee [1]. 

Coffee quality hinges on multiple aspects, from the variety and cultivation climate to the post-harvest processes like fermentation, drying, and roasting [2,3,4]. Natural fermentation can be achieved through dry/natural, wet, or semi-dry processing methods, each dictating the fermentation duration [5]. By harnessing region-specific microbial groups, the flavor profile of coffee can be enhanced, allowing for a diverse taste palette when the coffee fermentation happens worldwide. The most crucial part in this process is the role played by microbes in the development of aroma.

The wet processing method, despite its higher water consumption, stands out for its capacity to yield superior coffee, particularly suited for export. This process, from handpicking to storage, ensures consistent quality standards [6]. As coffee cherries ferment, they undergo a succession of microbial dominances, starting with bacteria in high-moisture settings, eventually giving way to yeast and fungi as the process concludes [7]. During fermentation, each microbe introduces plenty of metabolites, and understanding the microbial dynamics in wet fermentation is pivotal to unlocking coffee’s flavor secrets. The core aim is to expose the inner cellulose and starch structures of the coffee cherries by eliminating the polysaccharide-rich mucilage layer. As microbes feast on the cherries’ components, they produce growth metabolites that add to the coffee’s aroma, flavor, and bioactive compounds [5,8,9]. Yeast such as *Saccharomyces cerevisiae*, *Wickerhamomyces anomalus*, *Pichia kudriavzevii*, and *Papiliotrema flavescens* have been reported to produce pectinase enzyme that functions to complete digestion of pectin that consequently represents the positive effect on the coffee quality when fermenting the coffee beans among 28 field isolations [5]. This fermentation continues during drying, helping achieve the desired moisture level of less than 11%.

The advent of metagenomic techniques has revolutionized the understanding of coffee fermentation’s microbial ecosystems. Analyzing the total genetic content of all sample microorganisms has revealed the diversity and functional potential of these microbial communities. These methods illuminate the microbial groups that craft crucial flavor compounds, shaping the sensory attributes of brewed coffee. In coffee fermentation, the interplay among microbial communities significantly influences the end product. Factors such as metabolites, antimicrobial compound production, quorum sensing, and survival competition all converge to determine the taste of finished coffee. Furthermore, the enzymatic activities of these microbes can affect the coffee’s color [10]. Therefore, the role of bacteria in coffee flavor has drawn considerable attention. Several bacterial species generate enzymes that simplify complex molecules, releasing volatiles that enhance the coffee’s flavor. Their ability to modulate coffee’s acidity through organic acid production, combined with the microbial dynamics of fermentation, culminates in a broad spectrum of flavor profiles through the biochemical transformation in the final brew. For on-site coffee fermentation, safety hinges on meticulous sanitation. While naturally occurring yeast and lactic acid bacteria (LAB) in coffee cherries enrich the fermentation process, it is vital to ward off undesirable aromas arising from sources like enterobacteria, filamentous fungi, and other soil-dwelling microbial groups [11,12].

To create exotic coffee to serve the coffee industry and consumers, the aroma and metabolite profiles of coffee are highly impacted by the fermentation process. Therefore, in this research, we studied on-site coffee fermentation via metagenomics. We highlight the correlation between microorganisms in coffee fermentation using the wet process with coffee flavor, aroma and metabolites. Our metagenomic approach reveals insights into how specific microbial communities influence flavor, aroma, and metabolite determination. 

## 2. Materials and Methods

### 2.1. On-Site Wet Process Coffee Fermentation

Arabica Coffee (*Coffea arabica* L.) fruits were fermented by the wet process at the Organic Coffee Farm at Tongping, Chiang Mai, Thailand. The experiments were performed in triplicate during the harvesting seasons. A total of 60 kg of Arabica Coffee fruit was harvested at the mature stage (cherries), washed, and manually depulped, followed by 48 h fermentation in a tank with 60 L of water to remove the mucilage. At the farm, the temperature of fermentation was between 21 and 37 °C during the harvesting season. Fermentations were performed in triplicate while monitoring pH, moisture content, °Brix, and temperature. The initial sugar content of fruit (°Brix) was measured with a refractometer (Sigma-Aldrich, St. Louis, MO, USA). After fermentation, the coffee cherries were placed on suspended platforms for sun drying until they reached moisture levels of approximately 10% (144 h). Cherries were collected at 0 days (during the wet process fermentation), 2 days (during the wet process fermentation), 4 days (during the drying process), and 8 days (during the drying process) while the slurries were collected at 0 and 2 days. Samples (50 g) were placed aseptically in sterile falcon tubes, in triplicate, and immediately transferred to the laboratory in iceboxes for physicochemical analyses (volatile and metabolites). Coffee samples were frozen at −20 °C until analyzed. Slurries (20 mL) and cherries (20 g) were collected in DNA/RNA shields (Zymo Research, Irvine, CA, USA) at room temperature for the microbiome analysis through the metagenomic analysis.

### 2.2. Microbiome Sequencing and Bioinformatic Analysis of Bacterial and Fungal Communities

The samples were processed and analyzed with the ZymoBIOMICS^®^ Targeted Sequencing Service (Zymo Research, Irvine, CA, USA). The DNA extraction was performed by ZymoBIOMICS^®^-96 MagBead DNA Kit (Zymo Research, Irvine, CA, USA) using an automated platform. Bacterial 16S ribosomal RNA gene targeted sequencing was performed using the Quick-16S^TM^NGS Library Prep Kit (Zymo Research, Irvine, CA, USA). In most cases, the bacterial 16S primers amplified the V3–V4 region of the 16S rRNA gene. These primers have been custom-designed by Zymo Research to provide the best coverage of the 16S gene while maintaining high sensitivity. Fungal ITS gene targeted sequencing was performed using the Quick-16S^TM^ NGS Library Prep Kit with custom ITS2 primers substituted for 16S primers.

The sequencing library was created using an innovative library preparation process in which PCR reactions were performed in real-time PCR machines to control cycles and therefore limit PCR chimera formation. The final PCR products were quantified with qPCR fluorescence readings and pooled together based on equal molarity. The final pooled library was cleaned with the Select-a-Size DNA Clean & Concentrator^TM^ (Zymo Research, Irvine, CA, USA), then quantified with TapeStation^®^ (Agilent Technologies, Santa Clara, CA, USA) and Qubit^®^ (Thermo Fisher Scientific, Waltham, WA, USA). The ZymoBIOMICS^®^ Microbial Community Standard (Zymo Research, Irvine, CA, USA) was used as a positive control for each DNA extraction, if performed. The ZymoBIOMICS^®^ Microbial Community DNA Standard (Zymo Research, Irvine, CA, USA) was used as a positive control for each targeted library preparation. Negative controls (i.e., blank extraction control, blank library preparation control) were included to assess the level of bioburden carried by the wet-lab process. The final library was sequenced on Illumina^®^ MiSeq^TM^ with a v3 reagent kit (600 cycles). The sequencing was performed with 10% PhiX spike-in.

Unique amplicon sequence variants were inferred from raw reads using the DADA2 pipeline. Potential sequencing errors and chimeric sequences were also removed with the Dada2 pipeline. Taxonomy assignment was performed using Uclust from Qiime v.1.9.1 with the Zymo Research Database, a 16S database that is internally designed and curated, as referenced. Composition visualization, alpha-diversity, and beta-diversity analyses were performed with Qiime v.1.9.1. If applicable, taxonomy that had significant abundance among different groups was identified by LEfSe using default settings. Other analyses such as heatmaps, Taxa2ASV Deomposer, and PCoA plots were performed with internal scripts.

If performed, a quantitative real-time PCR was set up with a standard curve. The standard curve was made with plasmid DNA containing one copy of the 16S gene and one copy of the fungal ITS2 region prepared in 10-fold serial dilutions. The primers used were the same as those used in Targeted Library Preparation. The equation generated by the plasmid DNA standard curve was used to calculate the number of gene copies in the reaction for each sample. The PCR input volume was used to calculate the number of gene copies per microliter in each DNA sample. The resulting values are shown in the gene copies column of the absolute abundance results table.

The number of genome copies per microliter DNA sample (genome copies) was calculated by dividing the gene copy number by an assumed number of gene copies per genome. The value used for 16S copies per genome is 4. The value used for ITS copies per genome is 200. The amount of DNA per microliter DNA sample (DNA ng) was calculated using an assumed genome size of 4.64 × 10^6^ bp, the genome size of *Escherichia coli*, for 16S samples, or an assumed genome size of 1.20 × 10^7^ bp, the genome size of *Saccharomyces cerevisiae*, for ITS samples. This calculation is shown below:

Calculated Total DNA = Calculated Total Genome Copies × Assumed Genome Size (4.64 × 10^6^ bp) × Average Molecular Weight of a DNA bp (660 g/mole/bp) ÷ Avogadros Number (6.022 × 10^23^/mole)

### 2.3. Extraction and Analysis of Volatile Compounds Using Headspace Stir Bar Sorptive Extraction (HSSE) and Gas Chromatograph–Mass Spectrometer Triple Quadrupole (GCTQ)

Coffee cherries and slurry were analyzed using gas chromatography and mass spectrometry via triple quadrupole (Bruker model 436GC/EVOQ premium and Gerstel MPS robotic). The cherries were ground before being added to vials. A total of 0.5 g of ground cherries and 0.5 mL of the slurry were added to 20 mL vials, followed by addition of 5 mL of water (LC grade). The samples were sonicated for 5 min. The headspace stir bar sorptive extraction (HSSE) was used to extract the compounds from the cherry and the slurry samples at 70 °C, 700 rpm for 6 min using PDMS Twister to absorb the volatile compounds. The absorbed compounds on Twister were analyzed using the thermal desorption unit (TDU) (Mode: spitless, flow: 50 mL/min, ramp: 5 °C/min, end temperature 200 °C) and cooling injection system (CIS) (Mode: spitless, initial temperature 60 °C, flow 50 mL/min, ramp 10 °C/s, end temperature 200 °C, hold 1 min). The compounds were analyzed using GCTQ (model Bruker EVOQ premium, column BR 5, Pneumatics He; Pi 7.1 psi, constant flow 2.0 mL/min, oven = 60 °C, 2 min then increase to 200 °C and hold for 2 min, total runtime 15 min, MS mode full scan 35–550 da).

### 2.4. Biochemical Compound Analysis Using UHPLC

The UHPLC (Thermo Scientific, ultimate 3000) and mass spectrometer type quadrupole time-of-flight (QTOF) Bruker model compact (column: Bruker intensity solo2 C18 (100 × 2.1 mm)) was used for analysis of biochemical compounds; 0.5 g of ground cherries or 0.5 mL of slurry sample were added to 20 mL vials followed by addition of 5 mL of water (LC grade). The samples were sonicated for 5 min. The compounds were extracted using an agitator at 70 °C, 700 rpm for 6 min. The samples were filtered with nylon membranes (0.22 µm), then placed into 1.5 mL vials for UHPLC analysis. The UHPLC was operated with the sample injection volume of 5 µL, oven 35 °C, mobile phase (solution A: water, 5 mM ammonium formate, and 0.01% *v*/*v* formic acid; solution B: methanol, 5 mM ammonium formate, and 0.01% *v*/*v* formic acid) with the multiple-step gradient time of 0–12 min, flow rate of 0.30 mL/min, and MS with ESI 500V.

### 2.5. Data Analysis

The Spearman correlation coefficients between the flavor components/metabolites and the microbial taxa during coffee fermentation were calculated and visualized through Origin Pro 2022 software.

## 3. Result and Discussion

### 3.1. Metagenomic Analysis of Microbial Community during Coffee Fermentation

**Figure 1:**
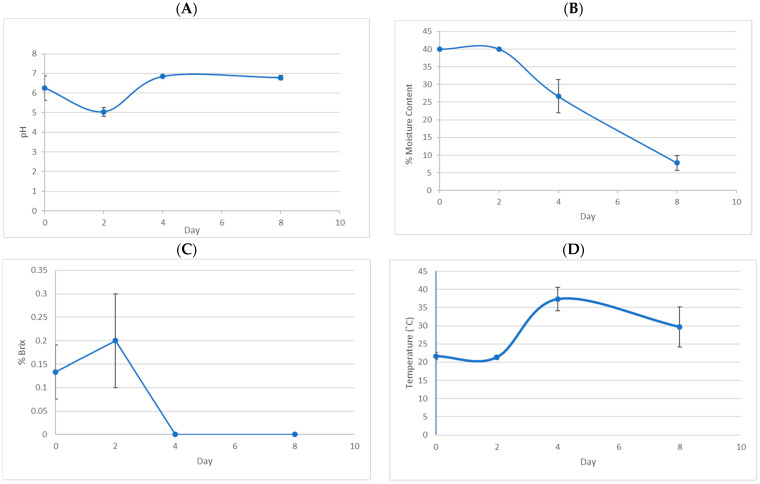
Characteristics of coffee during fermentation. (**A**) pH; (**B**) % Moisture content on wet basis; (**C**) % Brix; (**D**) Temperature at the site during the wet fermentation process (Days 0 and 2) and drying process (Days 4 and 8).

The environmental conditions also play a crucial role in the types of microorganisms present at different stages of fermentation, thereby affecting the quality of the final product. Five parameters are particularly important for this process, namely, sugar concentration (measured in °Brix), water activity (Aw), temperature, acidity, and time [7]. At the farm site, the environmental temperature varied between 21 and 37 °C during the harvesting season, fermentation, and drying process. Intact fruits were manually depulped and then fermented for 48 h in a tank with 60 L of water to remove the mucilage. Finally, the coffee was sun-dried until the beans reached approximately 10% moisture content. The initial ˚Brix value of cherries was approximately 0.14% and dropped to 0% within 4 days. pH dropped to 5 on Day 2 and rose to neutral toward the end of the process (Figure 1). 

**Figure 2:**
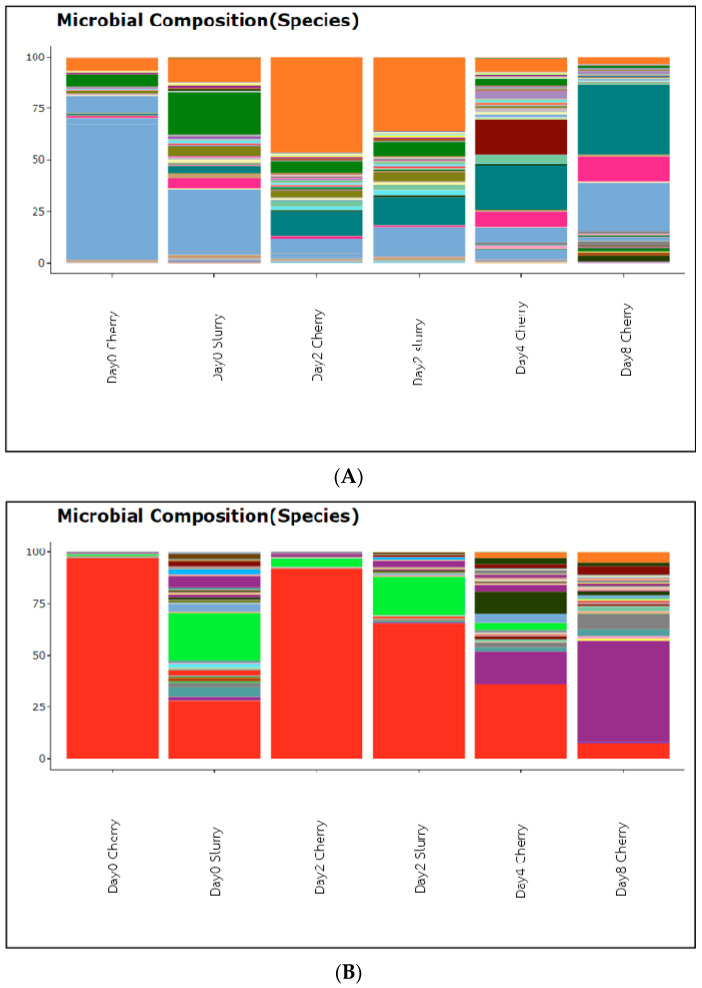
Taxa composition plots illustrate the (**A**) bacterial composition and (**B**) fungal and yeast composition at different taxonomy levels of species. Each color represented the specifically identified species.

**Figure 3:**
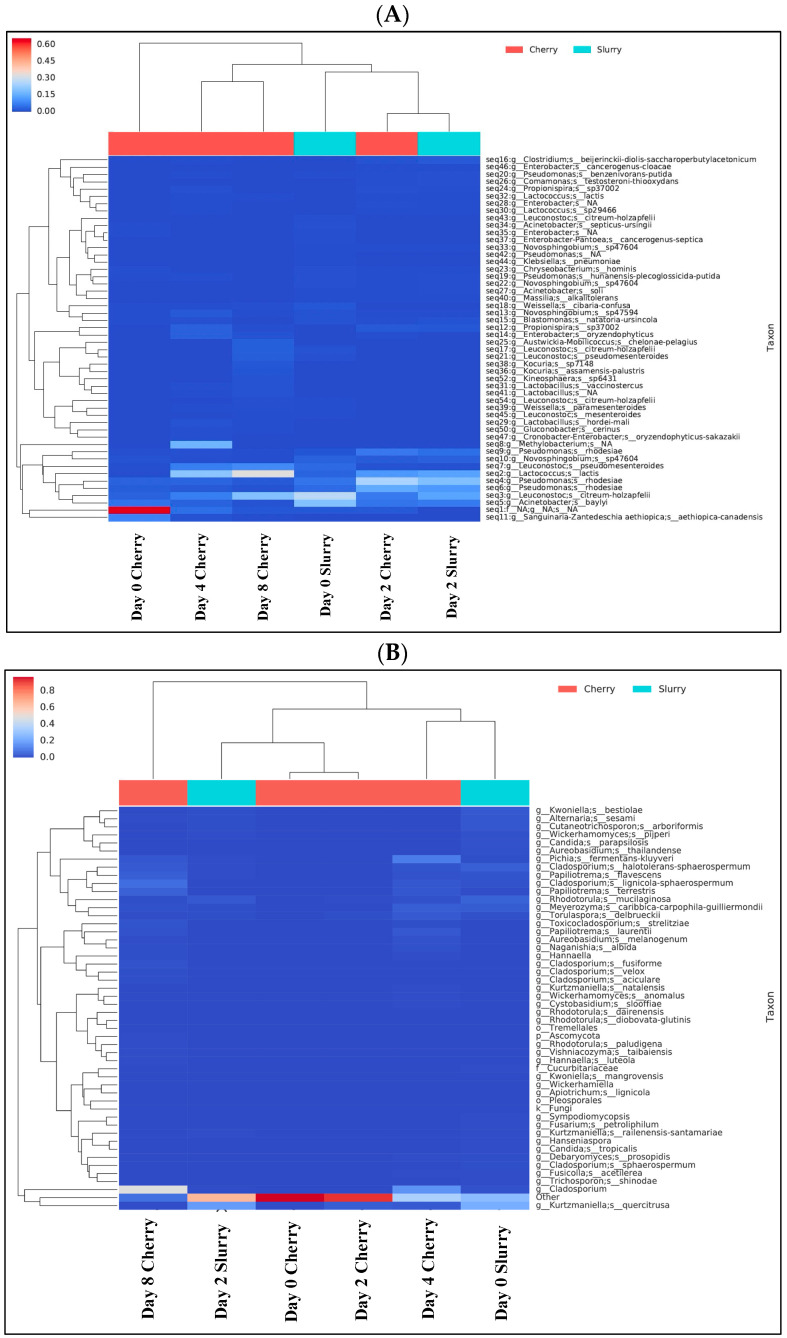
The taxonomy abundance heatmap with sample clustering is a quick way to help identify patterns of (**A**) bacterial and (**B**) fungal/yeast distribution among samples.

**Figure 4:**
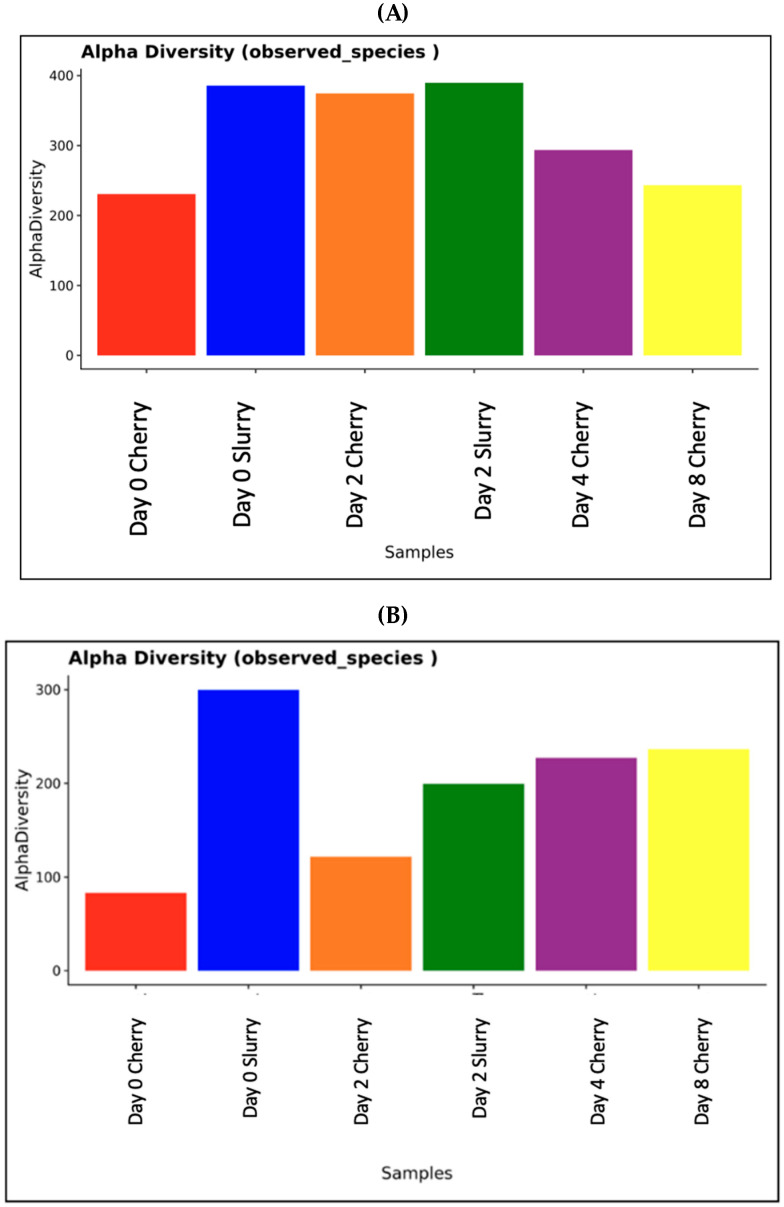
Alpha diversity was measured for (**A**) bacterial and (**B**) fungal and yeast diversity of each sample. The plot indicates the number of observed species in the samples. For analyses without group comparison, a histogram of observed species in each sample is shown.

Cherries were collected at Days 0, 2, 4, and 8, while the slurries were collected at Days 0 and 2. The absolute abundance of bacterial DNA was analyzed by 16S and fungal/yeast DNA was analyzed by ITS. Yeast and fungal DNA were highly abundant in the sample from Day 0 in cherries followed by Day 2 in cherries and slurry, while bacterial DNA was highly abundant on Day 2 in the slurry. Overall, the amount of the bacteria was higher than that of yeast and fungi. The diversities were less on Day 0 in cherries and highly diverse on Days 4 and 8 (Figure 2). The heatmap in Figure 3 shows the relative abundance of various bacterial and fungal genera among our six samples. We found that most genera of bacteria and fungi occur at low abundance. The samples had 64 genera of bacteria and 59 genera of yeast/fungi in common, however, they occurred in varying proportions between days of fermentation and drying. For bacterial communities, it was found that Day 2 cherry, Day 2 slurry, and Day 0 slurry were clustered, while Days 4 and 8 cherry were grouped together. For the fungal community, Day 4 cherry and Day 0 slurry had similar patterns of yeast/fungi, whereas Day 2 slurry, Day 0 cherry, and Day 2 cherry were clustered. The ∝-diversity was determined to assess the species richness. Figure 4 illustrates the diversity observed among the samples. In cherries, the bacterial diversity increased from Day 0 to Day 2 and gradually decreased on Day 4 toward Day 8. In the slurry, there was a great richness of bacterial communities on both Day 0 and Day 2. The yeast and fungal diversity in the cherry groups increased throughout the fermentation and drying periods. However, it decreased from Day 0 to Day 2 in the slurry group. To evaluate the microbial diversity between the two groups, a peer comparison of the abundance between the cherry and slurry was carried out. The whiskers in the whisker box plot show the range of minimum and maximum ∝-diversity values within a population. The data indicated that the slurry had more microbial abundance than cherries. Therefore, in the wet process of field fermentation, it is important to leave the samples submerged for 2 days without any water replacement in order to maintain the number and variety of fermentative microbes. 

**Figure 5:**
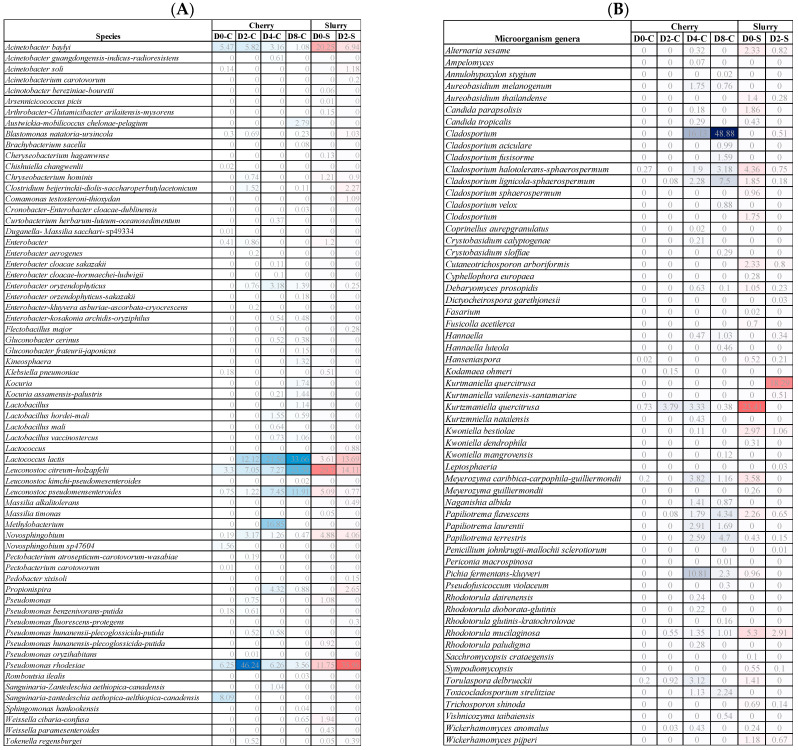
Dynamics of microbial communities during fermentation: (**A**) Bacteria; (**B**) Fungi and Yeast. Cherry samples were collected at Days 0, 2, 4, and 8 and denoted as D0-C, D2-C, D4-C, and D8-C, respectively. Slurry samples during fermentation were taken at Day 0 (D0-S) and 2 (D2-S). The numbers represent the % coverage of each species, the intensity represents the amount while blue and red represent the comparison for cherry and slurry, respectively.

This study characterized the dominant microbial communities of bacteria and fungi present in wet fermentations through a metagenomic approach and, further, the biochemical compounds (organic acids, bioactive constituents, and volatiles) generated by these communities. During fermentation, microorganisms consume carbohydrates or other organic compounds and proliferate. Most microorganisms that participate in the process come from the environment (soil, air, plants, and other sources) [7]. In wet processing, after harvesting and pulping, the coffee beans are submerged in masonry tanks containing large volumes of water. The microaerophilic environment and nutritional selectivity of coffee pulp favor the growth of microorganisms with fermentative metabolism, mainly yeast, fungi, and bacteria. Microbial activities degrade the components of mucilage (simple sugars, complex carbohydrates, and proteins) and induce the biochemical transformations necessary for natural fermentation. From our results, yeast from the generas *Pichia*, *Kurtzmaniella*, and *Cladosporium* sp. and bacteria belonging to *Leuconostoc* sp., *Acinetobacter* sp., *Lactococcus* sp., *Lactobacillus* sp., *Novosphingobium* sp., *Pseudomonas* sp., *Enterobacter* sp., and *Weissella* sp. were often found during this on-site wet fermentation process (Figure 5). *Lactococcus lactis*, *Leuconostoc citreum-holzapfelii*, *Leuconostoc pseudomensenteroides*, *Pseudomonas rhodesiae*, and *Acinetobacter baylyi* were predominant during fermentation in both samples and remained in the cherry samples until Day 8. In these cherry samples, *Lactococcus lactis*, *Leuconostoc citreum-holzapfelii*, and *Leuconostoc pseudomensenteroides* continuously increased until Day 8, while *Acinetobacter baylyi* and *Pseudomonas rhodesiae* decreased during the fermentation process. This correlates with the pH of the fermentation condition, which dramatically dropped at the beginning of the fermentation period. The presence of both *Pseudomonas rhodesiae* and *Lactococcus lactis* was increased in the slurry and cherries after 2 days of the process. In addition, *Acinetobacter soli*, *Blastomonas natatoria-ursincola*, *Clostridium beijerinckii-diolis-saccharoperbutylacetonicum*, *Comamonas testosteroni-thioxydan*, *Novosphingobium* sp., and *Propionispira* sp. were found at levels above 1% in the second day of the fermentation process. 

In terms of yeast and fungi, there was a significant increase in *Cladosporium* sp., *Cladosporium halotolerans-sphaerospermum*, *Cladosporium lignicola-sphaerospermum*, *Papiliotrema flavescens*, *Papiliotrema laurentii*, and *Papiliotrema terrestris* in cherries by Day 8. Additionally, *Pichia fermentans-kluyveri* was the second most dominant at Day 4 of fermentation and then decreased by Day 8. In slurry, high levels of *Kurtzmaniella quercitrusa*, *Cladosporium halotolerans-sphaerospermum*, *Cladosporium lignicola-sphaerospermum*, *Alternaria sesame*, *Candida parapsolisis*, *Cutaneotrichosporon arboriformis*, *Meyerozyma caribbica-carpophila-guilliermondii*, and *Rhodotorula mucilaginosa* were found on Day 0 and declined afterward. *Kurtmaniella quercitrusa* was dominant on the second day of the fermenting process. *Candida tropicalis* was the most prevalent in cherries toward the end of the fermentation process. *Kurtzmaniella quercitrusa* is a yeast that was found in amounts of up to 23.41% in the fermentation process, which is interesting because this is a xylose utilizing microbes and xylose is only present in tiny amounts in the coffee bean [13]. From our study, *Pichia fementans-kluyveri* was the dominant yeast in coffee fermentation (up to 10.81%), followed by *Rhodotorula* sp. (5.3%) and *Torulaspora* sp. (3.12%). Previous studies showed that *Pichia anomala*, *Torulaspora delbrueckii*, and *Rhodotorula mucilaginosa* were the dominant yeasts in coffee *Coffea arabica* fermentation in Brazil [14]. In agreement with that study, we found *Rhodotorula mucilaginosa* was one of the dominant yeasts in coffee fermentation (between 1.0 and 5.3%). This yeast has been reported to produce carotenoids [15]. Furthermore, *Rhodotorula mucilaginosa* has been reported as a biological control agent to inhibit or reduce sporulation and mycelial growth by competing for space and nutrients. Additionally, this yeast exhibits antibiosis and direct parasitism by producing VOCs, killer toxins, and lytic enzymes (proteases, chitinases, and glucanases) that prevent contamination of natural coffee beans. For these reasons, *Rhodotorula mucilaginosa* has been applied as a biological agent in field conditions [16]. Other studies have shown that the most common bacteria genera present during coffee fermentation are *Bacillus* sp., *Lactobacillus* sp., *Acinetobacter* sp., *Arthrobacter* sp., *and Weissella* sp. and yeasts are *Pichia* sp., *Saccharomyces* sp., *Rhodotorula* sp., *Candida* sp., *Kluyveromyces* sp., and *Hanseniaspora* sp. [7]. Based on our results, the bacterial community seemed to initiate the fermentation process on-site earlier than yeast and fungi. However, all of these microbes play various roles in the fermentation process, especially those yeast and bacteria that degrade mucilage (pectinolytic activity), inhibit mycotoxin-producing fungal growth, and produce flavor-active components.

Studies have shown that *Pseudomonas* sp. isolated from *Coffea arabica* can produce siderophores and hydrogen cyanide (HCN) and exhibits remarkable inhibition against emerging fungal coffee pathogens to biologically control coffee wilt diseases [17]. From our study, *Pseudomonas* sp. was found toward the end of the fermentation period. *Novosphingobium* sp. has been reported to degrade aromatic compounds from lignocellulosic material and these bacteria were found in a significant amount in our slurry samples [18]. *Chishuiella* sp. is one of the key microbial factors for higher ethanol production and higher dry mass loss in silage fermentation containing extensive C3 and C4 sugars [19]. *Klebsiella pneumonia* is a pectolytic bacteria commonly found in the fermentation of *Coffea arabica* and produces pectate lyase with optimal activity at pH 8 [3]. The pectolytic microorganisms exert microbiological control of the fermentation but do not enhance the rate of fermentation, while acidification from lactic acid bacteria is important to accelerate the fermentation process [3]. *Pectobacterium carotovorum* has been reported to produce pectate lyase (PL, EC 4.2.2.2) for penetration into the plant cell wall leading to maceration of plant tissues [20]. We found *K. pneumonia* and *P. carotovorum* in small amounts on Day 0 in cherry and slurry samples and suggest they may be essential to degrade pectin for further fermentation processes. *Enterobacter* sp. is one of the predominant microbes in coffee fermentation. *Weissella* sp., which is phylogenetically related to *Leuconostoc* sp, was found at significant levels in slurry in the initial stages. Some strains of *Weissella* sp. have been reported to have probiotic properties and have been isolated from a wide variety of fermented food products (including those of vegetable origin, sourdough, kimchi, fermented soya, and breast milk), as well as being part of the normal microbiota of human intestines [21,22,23,24,25,26]. Heterofermentative *Leuconostoc* sp., *Lactococcus lactis*, *Enterococcus* sp., and *Weissella* sp. were the most common LAB found in fresh coffee cherries in Taiwan, as well as in our study. They produce bacteriocin-like inhibitory substances that are potential antagonists of foodborne fungi in coffee fermentation [27]. *Leuconostic citreum* appeared in cherry and slurry samples throughout the fermentation period and has been reported to produce high levels of ketones and ethanol in fermented products [28]. *Lactobacillus* sp., *Acetobacter* sp., *Leuconostoc* sp., *Lactococcus* sp., *Novosphingobium* sp., *Blastomonas* sp., *Acinetobacter* sp., *Weissella* sp., and *Springomonas* sp. have been found as mixed cultures in the production of volatile compounds in fermented rice noodles [29]. All of these were found in our study in differing amounts from Day 0 to Day 8 and probably played significant roles in developing the flavor and aroma compounds in coffee. We found various lactic acid bacteria at the fermentation site and in the cherry samples. Production of lactic acid results in decreasing pH, which was most apparent in our study on Day 2 (Figure 1A, Table 1). The lactic acid, in turn, assists in the breakdown of pectin, a complex carbohydrate present in high concentrations in coffee pulp. Their metabolic activities aid in the subsequent drying process by eliminating coffee mucilage constituents, causing a reduction in the time required for post-harvest processing from 21 days to 7 days [3,30,31]. LAB have been associated with the generation of bioactive compounds related to coffee quality. The flavor-forming capability of ‘wild-type’ LAB cultures in coffee fermentation has gained increased interest due to the diverse aromas such strains are capable of imparting. Some LAB-derived metabolites, such as esters, ketones, higher alcohols, and aldehydes, can influence sensory attributes of coffee beverages with distinct floral, fruity, and buttery perceptions [32,33]. In addition, *Lactobacillus* sp. and *Leuconostoc* sp. have been reported to produce enzymes such as pectinases and xylanases to depolymerize polysaccharides, in addition to producing lactic acid to promote yeast growth on the depolymerized media [3].

Some microbes commonly found in the environment in the fermentation process, such as *Chryseobacterium* sp., are Gram-negative non-fermentative microbes that are potentially pathogenic [43]. *Pedobacter* sp., which was found in trivial amounts in our fermentation field, is associated with the production of vinegar and is correlated with flavor metabolite productions during different stages of acetic acid fermentation [44]. *Methylobacterium* sp. is positively correlated with the amount of succinic and citric acid in coffee fermentation and had a relative abundance of 16.85% in the cherry samples on Day 4 [45]. The acetic acid bacterium, *Gluconobacter cerinus*, has been isolated from grapes and metabolizes ethyl alcohol into acetic acid in the fermentation process [46]. We found only a small amount of *Gluconobacter cerinus* in our fermentation field. *Propionispira* was quite predominant in the middle period of the process. It has been reported to produce end products such as propionic acid, acetic acid, and carbon dioxide, while ethanol and succinate are formed under special conditions [47]. *Kocuria* sp. is found in fermented food ingredients and has antimicrobial properties to control the growth of *Bacillus cereus* [48]. *Kocuria* sp. has also been reported to produce hydrogen from coffee residues, including mucilage, and was found in cherries in our study on Day 4 [49]. *Curtobacterium* sp. are a group of bacteria with unknown function that are found at high levels in berry tissue, especially coffee cherries, that found in our cherry samples [50]. Some other bacteria found in our fermentations in small amounts have been reported with other functions. *Brachybacterium* sp. has been found to produce acid from various sugar sources when isolated from salted fermented seafoods [51]. *Romboutsia* sp. has been reported as a hydrogen-producing bacteria isolated from anaerobically digested sludge [52]. *Romboutsia* sp. was also identified as one of the gut microbiota with a positive correlation to attenuate metabolic syndrome in a coffee consumption study in rats [53]. 

### 3.2. The Correlation between the Microbial Community and the Developed Flavor/Aroma

**Figure 6:**
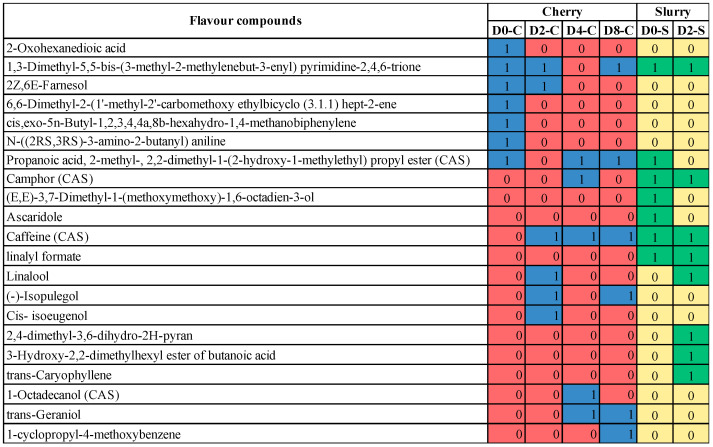
Changes in flavor compounds during fermentation where the number 1 indicates flavors detected in cherry (blue) and slurry (green), while the number 0 indicates flavors that were not detected in cherry (red) and slurry (yellow).

**Figure 7:**
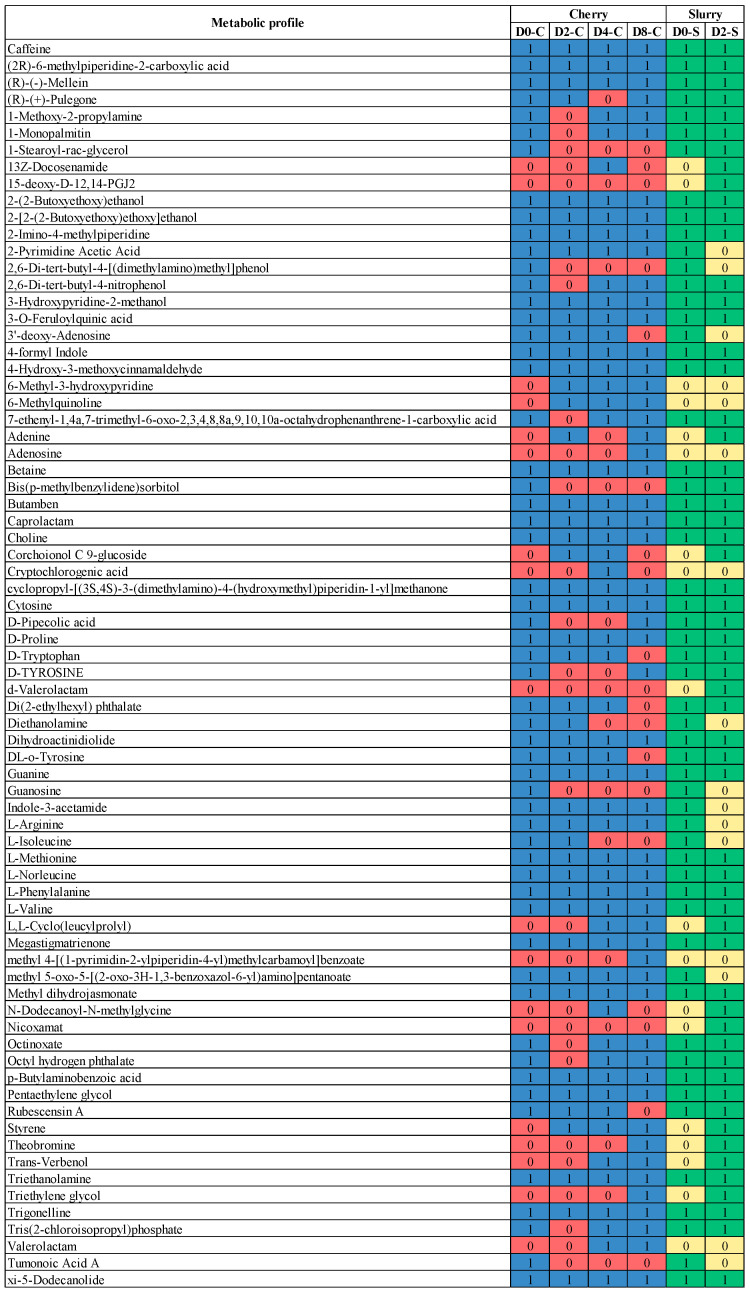
Changes in metabolic profile during fermentation. Compounds that were detected are denoted with the number 1 for cherry (blue) and slurry (green), while the number 0 indicates compounds not detected in cherry (red) and slurry (yellow).

Microbial communities change in response to environmental conditions and the location where fermentations are carried out can affect coffee quality. Those conditions include temperature, moisture, and altitude [54]. *Gluconobacter* sp. and *Weissella* sp. are dominant in coffee fruits from altitudes of 800 to 1000 m. Among the Eukaryotic community, yeasts are the most dominant in all altitudes [45]. Because geological differences can affect the quality of coffee products based on the different types of the microbial communities present, the use of starter cultures (mainly yeast strains) has emerged in recent years as a promising alternative to control the fermentation process and to promote quality development of coffee products. However, the microbes present in the field are still an essential factor that influences the aroma of the coffee [55]. In our study, we found a correlation between the microbial community and biochemical compounds in terms of aroma, flavor, and metabolites (Figure 6 and Figure 7 and Table 1). Fermentation led to the change in flavor/aroma compounds in both cherry and slurry. As shown in Figure 6, some flavor compounds in cherry such as 1,3-Dimethyl-5,5-bis-(3-methyl-2-methylenebut-3-enyl) pyrimidine-2,4,6-trione and propanoic acid remained after processing. Some new compounds were produced during processing, including caffeine, linalool, (-)-Isopulegol, cis-isoeugenol, 1-octadecanol (CAS), trans-geraniol, 1-cyclopropyl-4-methoxybenzene, camphor (CAS); however, only caffeine (CAS), (-)-isopulegol, and trans-geraniol were still detectable on cherries in the last stage of the process. In slurry samples, the flavor compounds such as 1,3-dimethyl-5,5-bis-(3-methyl-2-methylenebut-3-enyl) pyrimidine-2,4,6-trione, caffeine, linalyl formate, and camphor (CAS) were found to be produced from Day 0 to Day 2 in slurry and, with the exception of camphor, remained in cherries until Day 8. The production of linalool, 2,4-dimethyl-3,6-dihydro-2H-pyran, 3-hydroxy-2,2-dimethylhexyl ester of butanoic acid, and trans-caryophyllene were also detected in the fermentation process. Figure 7 shows the overall metabolites that were found in the fermentation process. Most of the metabolites that were produced in the initial phases of the fermentation process were present until the end, with the exception of caprolactam, 1-Stearoyl-rac-glycerol, 13Z-docosenamide, 15-deoxy-D-12,14-PGJ2, 2,6-di-tert-butyl-4-[(dimethylamino)methyl] phenol, Bis (p-methylbenzylidene) sorbitol, corchoionol C 9-glucoside, cryptochlorogenic acid, D-Tryptophan, d-Valerolactam, Di(2-ethylhexyl) phthalate, DL-o-Tyrosine, Guanosine, L-Isoleucine, N-Dodecanoyl-N-Methylglycine, rubescensin A, and Tumonoic acid A. Additionally, new compounds were found during the fermentation of coffee cherries, such as 6-methyl-3-hydroxypyridine, 6-methylquinoline, adenine, adenosine, caprolactam, L,L-cyclo(leucylprolyl), N-dodecanoyl-N-methylglycine, styrene, theobromine, *trans-*verbenol, triethylene glycol, and valerolactam.

**Figure 8:**
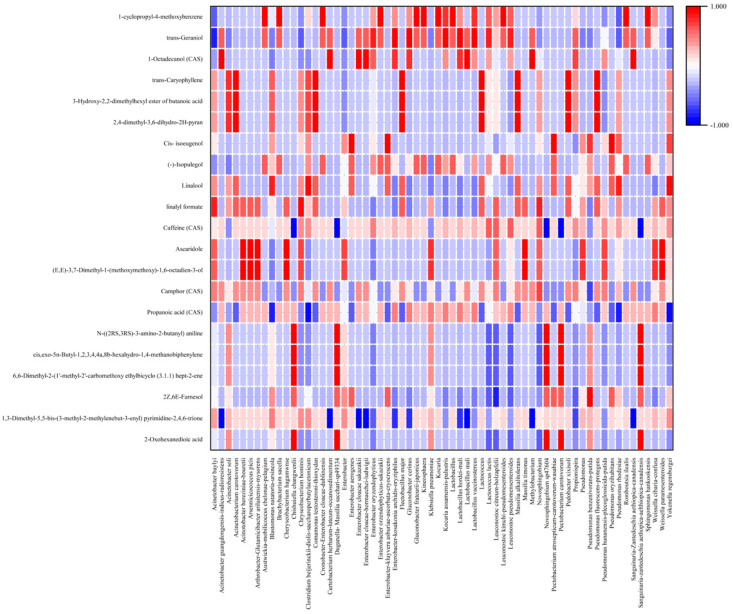
Heatmap of flavor profiles and bacteria at different fermentation stages analyzed by Spearman’s correlation.

**Figure 9:**
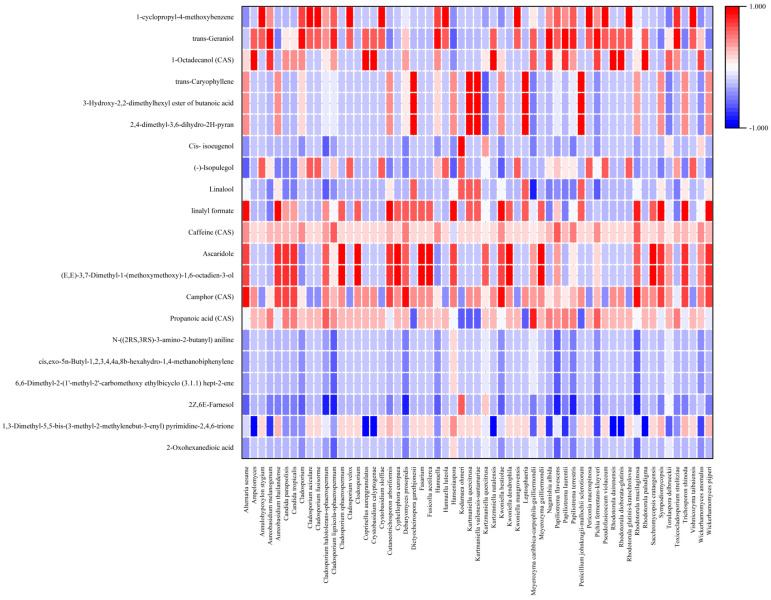
Heatmap of flavor profiles and yeast/fungi at different fermentation stages analyzed by Spearman’s correlation.

The correlation between microbial profile and flavor/aroma compounds is shown in Figure 8 and Figure 9. Camphor (CAS), mainly of Camphoreous aroma, was positively correlated with yeast/fungi such as *Alternaria sesame*, *Debaryomyces prosopidis*, *Kwoniella bestiolae*, and *Rhodotorula mucilaginosa* (*p* > 0.7) [56]. Camphor is associated with woody, soapy, and fatty aromas and appears mainly when lactic acid bacteria are not predominant. Camphor has been reported to have anti-tumor activity in mice [57]. Compounds of linalyl formate produce aromas of citrus, herbal, bergamot, and lavender and are associated with three types of bacteria (*Acinetobacter baylyi*, *Chryseobacterium hominis*, *Novosphingobium* sp.) and nine types of yeast/fungi (*Alternaria sesame*, *Aureobasidium thailandense*, *Cutaneotrichosporon arboriformis*, *Hanseniaspora*, *Kwoniella bestiolae*, *Rhodotorula mucilaginosa*, *Sympodiomycopsis* sp., *Trichosporon shinoda*, *Wickerhamomyces pijperi*). Linalool, with aromas of citrus, floral, sweet bois de rose, and blueberry was negatively correlated with *Meyerozyma caribbica-carpophila-guilliermondii*. However, it was remarkably positively correlated with bacterial species *Blastomonas natatoria-ursincola*, *Clostridium beijerinckii-diolis-saccharoperbutylacetonicum*, *Pseudomonas rhodesiae*, and *Yokenella regensburgei*. Cis-isoeugenol, with aromas of sweet spicy, carnation, and floral, was only positively correlated with one type of yeast (*Kodamaea ohmeri*) and six types of bateria, which mainly belong to the family of *Enterobacteriaceae* sp., *Pectobacterium* sp., and *Pseudomonas* sp. Trans-geraniol, that produces sweet floral, fruity, rose, and citrus aromas, was positively correlated with *Aureobasidium melanogenum*, *Cladosporium* sp., *Clalignicola-sphaerospermum*, *Hannaella* sp., *Naganishia albida*, *Papiliotrema laurentii*, *Papiliotrema terrestris*, *Pichia fermentans-kluyveri*, *Enterobacter* sp., *Gluconobacter* sp., *Kocuria* sp., and lactic acid bacterias. However, a remarkable negative correlation was found between trans-geraniol and *Acinetobacter baylyi* (reported *p* value of −0.828). The compound (-)-isopulegol is associated with a minty cooling flavor, but was not correlated with *Pichia fermentans-kluyveri*, *Enterobacter* sp., *Leuconostoc pseudomensenteroides*, or *Pseudomonas rhodesiae* in this study. However, it was also not significantly affected by any type of microbes (−0.7 < *p* < 0.7).

It has been reported that certain fungi present during coffee fermentation play a significant role in the production of pectin lyase, which catabolizes the 1,4-glycosidic links of fully esterified (methoxylated) chains of pectin. *Cladosporium* sp., which produces both pectinase and cellulase, was the most abundant yeast isolated from our samples of fermented coffee, with up to 48.88% in cherry samples on Day 8 [7]. Most of the fungi present in coffee fermentation are known as producers of large numbers of volatile compounds (2,4,6 trichloroanisole (TCA), geosmin, terpmonoterpenes, sesquiterpenes, alcohols, aldehydes, aromatic compounds, esters, furans, hydrocarbons, and ketones). Filamentous fungi also produce nitrogen and sulfur compounds, which are formed during primary and secondary metabolism and are associated with off-flavor development. Some *Penicillium* strains produce flavors that are more positive in sensory evaluations (floral aromas, caramels, sweet, slightly astringent aroma). We found a significant correlation on Day 4 in cherries between the appearance of *Pichia fementans-kluyveri* and the fruity aroma of trans-geraniol (Figure 5 and Figure 9). Beverages from the beans fermented by *Pichia* sp. are characterized by flavor attributes such as vanilla and floral aromas [58]. *Saccharomyces* sp., *Pichia* sp., and *Candida* sp. have been reported to produce volatile compounds (such as acetaldehyde, ethanol, ethyl acetate, and isoamyl acetate) which impart desirable attributes such as caramel, fruity, and buttery flavors that are characteristic of individual yeast starter cultures [59,60]. In our study, *Penicillium* was significantly correlated with the formation of linalyl formate and linalool and slightly correlated with camphor. Linalool and linalyl formate have been reported to produce a fruity, floral, and herbal aroma. *Torulaspora delbrueckii* is a yeast with a wide habitat, including on varieties of fruit, that is a promising biotechnological model to be exploited in a wide range of industries [61]. It produces an organoleptic profile with flavor- and aroma-enhancing properties when used in wine, beer, or bread dough fermentation [62,63]. Co-cultures using *Saccharomyces cerevisiae* and *T. delbrueckii* species produced 47 volatile compounds (predominately alcohols, aldehydes, and esters) which enhanced bread quality, imparting superior aroma and improved sensorial attributes [64]. However, we did not find any significant correlation between *Torulaspora delbrueckii* and aroma produced in our coffee fermentation. *Hanseniaspora uvarum* is a yeast that has been isolated in both coffee and cocoa fermentations. It has been used as a starter culture to create flavor and aroma in controlled coffee fermentations and exhibits pectinolytic activity and adaptability to stress conditions [65,66,67]. In our study, we found a correlation between the production of linalyl formate and the presence of *Hanseniaspora uvarum*. While *Alternaria* is considered a field fungi that contaminates coffee fruit, it can also positively influence flavor and aroma by the production of camphor and linalyl formate, as found in our study [68]. Fruity flavor compounds (apple, cherry) are produced in samples inoculated with *Candida parapsilosis*, and therefore, it has been used as a yeast starter culture in fermented coffee and in the production of flavor compounds from olive mill waste [69,70]. *Candida tropicalis* was present in our fermentation at 0.43% and we found a strong correlation with camphor and a slight correlation with linalyl formate. *Meyerozyma guilliermondii* has been reported to stimulate the multiplication of lactic acid bacteria. *Wickerhamomyces anomalus* was present at 0.03% in our fermentation. It has been reported to produce a high total phenol content (TPC) and flavonoid content (TFC) when used as a starter culture. Additionally, the compounds it produces have antioxidant activity and have been shown to improve the tastes and flavors of fermented coffee [71]. *Sacchromycopsis crataegensis* is a yeast that creates aroma and flavor precursors in fermented cocoa [63,72]. In our study, there was a correlation between *Sacchromycopsis crataegensis* and the production of camphor and linalyl formate. *Papiliotrema flavescens*, found in our fermentation at 4.34%, has been reported as a pectinolytic yeast used as a starter culture for coffee fermentation during wet processing and provides positive results on coffee quality [5]. We found that it significantly contributed to the production of trans-geraniol. *Torulaspora delbrueckii* (present at 1.41% in our fermentation) has gained attention in biotechnology for its flavor- and aroma-enhancing properties in various fermentation products. It has been shown to produce 47 volatile compounds, predominantly alcohols, aldehydes, and esters, which improve sensory attributes [64]. *Aureobasidium thailandense* was present at 0.28% in the fermentation and was highly correlated with the production of linalyl formate and camphor. Β-glucan from *A. thailandense* is a prebiotic that exhibits an immense potential in the food industry with wide applications as a functional ingredient and texturing agent [73]. *Wickerhamomyces pijperi* was found at a small percentage in our coffee fermentation and has been reported to be involved in cocoa fermentation in Brazil [74]. *Rhodotorula*, which was found in small amounts in our fermentation, has been isolated from permanent cold habitats and has the ability to produce cold-active pectinases [75]. *Annulohypoxylon stygium*, present in a tiny amount of 0.02% in our fermentation, is described for extremely high performance in lignin and carbohydrate degradation [76]. *Aureobasidium melanogenum* (found at 1.75% in our fermentations) is reported to produce tannase as a potential enzyme for food and agricultural processing [77]. Tannase is used as a clarifying agent in some wines, fruit juices, and refreshing drinks, as well as in developing a coffee flavor [78]. *Acinobacter* sp., *Leuconostoc* sp., and *Lactococcus* sp. were present at a high percentage in the fermented coffee on Days 0, 2, 4, and 8 and correlated with citrus aroma (Figure 5). Lactic acid bacteria have been shown to play important roles in citric acid metabolism and the synthesis of esters such as diethyl succinate esters (fruity aroma) [46]. From our results, lactic acid bacteria also correlated with the production of trans-geraniol, (-)-isopulegol, and linalyl formate, a group of compounds that are linked with floral and fruity aromas. Other microbes of unknown function were also present in the fermentation site and the impact on coffee quality is still unclear and controversial [6]. Therefore, based on these correlations, a future prospective would be to use starter cultures to impart specific flavors and aromas to coffee.

### 3.3. Metabolic Profiles Produced by the Microbial Community in Coffee Fermentation

**Figure 10:**
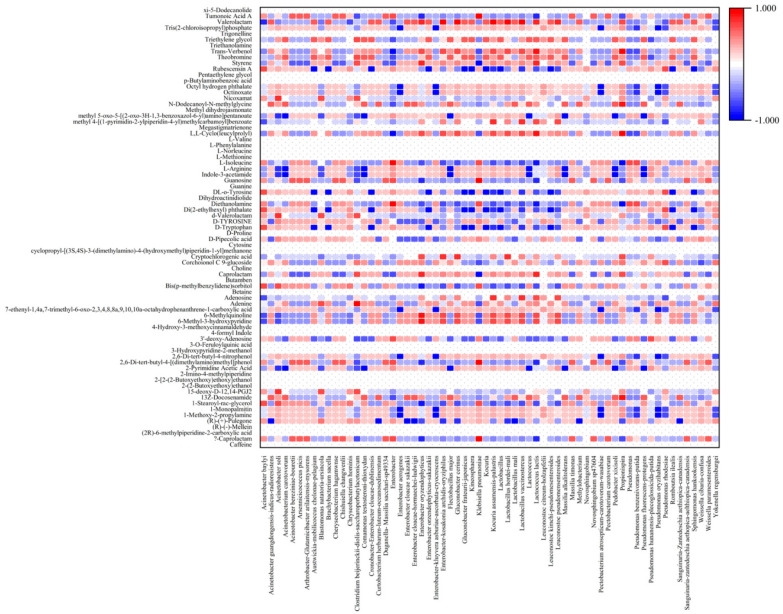
Heatmap of metabolite profiles of bacteria at different fermentation stages analyzed by Spearman’s correlation.

**Figure 11:**
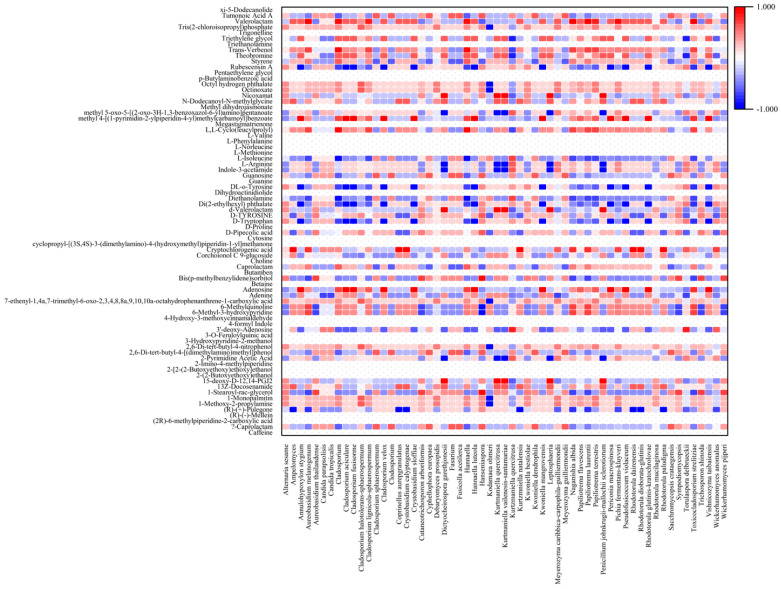
Heatmap of metabolite profiles of yeast/fungi at different fermentation stages analyzed by Spearman’s correlation.

Coffee brewed from green coffee beans is characterized by non-coffee-like aromas, such as musty/earthy and green and citrus flavors, which create an off-balance flavor with negative value for consumers. The changes in the chemical composition of green coffee beans is brought about by metabolic activities that occur during the course of coffee processing [79]. Metabolites fermented by microbes diffuse into the seeds, which improves their quality. In our study, coffee cherries were wet processed and dried to produce green beans which were then analyzed by liquid chromatography–mass spectrometry (LC–MS) to ascertain the metabolic profiles (Figure 10 and Figure 11). The presence of *Acinetobacter soli*, *Acinetobacterium carotovorum*, *Clostridium beijerinckii-diolis-saccharoperbutylacetonicum*, *Comamonas testosteroni-thioxydan*, *Flectobacillus major*, *Lactococcus* sp., *Pedobacter xixisoli*, *Pseudomonas hunanensis-plecoglossicida-putida*, *Dictyocheirospora garethjonesii*, *Kurtzmaniella quercitrusa*, *Kurtzmniella natalensis*, *Leptosphaeria* sp., and *Penicillium johnkrugii-mallochii sclerotiorum* resulted in increased production of 2,4-dimethyl-3,6-dihydro-2H-pyran, 3-Hydroxy-2,2-dimethylhexyl ester of butanoic acid, and trans-Caryophyllene. In addition, 1-Octadecanol (CAS), trans-Geraniol, and 1-cyclopropyl-4-methoxybenzene were postively correlated with *Enterobacter* sp., lactic acid bateria, and some types of yeast and fungi. Some compounds, such as valerolactam, were positivity correlated with groups of microorganisms (*Lactococcus lactis*, *Leuconostoc pseudomensenteroides*, and most fungi/yeast) and negatively correlated with others (*Acinetobacter baylyi*, *Pseudomonas rhodesiae*). The lactic acid bacteria genus also positively correlated with some compounds, such as 6-methyl-3-hydroxypyridine, 6-methylquinoline, styrene, theobromine, *trans-*verbenol, and triethylene glycol (*p* > 0.7). During the fermentation process, an increasing load of *Cladosporium* sp. positively correlated with some metabolites such as adenosine, methyl 4-[(1-pyrimidin-2-ylpiperidin-4-yl) methylcarbamoyl]benzoate, theobromine, triethylene glycol, and valerolactam. In addition, *Acinetobacter soli* was positively correlated with 15-deoxy-D-12,14-PGJ2, d-valerolactam, and nicoxamat. It was also found that *Kurtmaniella quercitrusa* was the main producer of 15-deoxy-D-12,14-PGJ2 and d-Valerolactam Nicoxamat. Fermentation by yeast and fungi mostly affected the levels of 1-Monopalmitin, 1-methoxy-2-propylamine, octinoxate, and octyl hydrogen phthalate. These metabolic compounds found in our coffee fermentation have been reported as functional active compounds. The expression of proteases, isocitrate lyase (ICL), and β-tubulin peaked during the fermentation process in wet processing and led to the germination of coffee beans and an increase in the concentrations of free amino acids [80]. We found L-Arginine, L-Valine, L-methionine, L-isoleucine, L-phenylalanine, D-tryptophan, D-tyrosine, and D-proline were increased beginning on Day 2. Caffeine, a methylxanthine that is heat stable and has bitter sensory characteristics, stimulates the central nervous system and is most commonly ingested through consumption of coffee, tea, and soft drinks [36,81]. Butamben can function as a nerve block to treat chronic pain of cancer and noncancer origin and has been found in coffee fermentations [82,83]. Betaine has anti-inflammatory functions in several human diseases such as obesity, diabetes, cancer, and Alzheimer’s disease [84]. Trigonelline has therapeutic activities in diabetes, diabetic complications, and central nervous system disease [85]. Triethanolamine has been reported to possess antibacterial and antifungal activities [86]. Guanosine is a purine nucleoside with important functions in cell metabolism and a protective role in response to degenerative diseases or injury and with a positive effect on the central nervous system [87]. 3’-deoxy-Adenosine and methyl dihydrojasmonate, which were found in our metabolic analysis, have been reported to have anti-tumor effects [88,89]. R-(+)-Pulegone is allergen suppressive and anti-inflammatory [90]. Choline is produced during fermentation and is an essential nutrient with roles in neurotransmitter synthesis, cell-membrane signaling, lipid transport, and brain and memory development in the fetus [91]. Methyl dihydrojasmonate has been reported to have an anticancer effect [89]. Some metabolites found in the coffee fermentation process, such as choline, betaine, trigonelline, Methyl dihydrojasmonate, 3′-deoxy-Adenosine, cryptochlorogenic acid, and, L-Cyclo(leucylprolyl), are essential for neurodevelopment and reduce the risks of cardiovascular disease and diabetes, while also possessing antibacterial and antitumor activities [85,88,89,92,93,94]. Theobromine is found in cocoa at high levels and was also present in our coffee fermentation. This compound has a pleasant flavor and is useful as an anti-inflammatory in asthma and other respiratory tract problems [95]. Some metabolite compounds from our study have been reported to have antioxidant and anti-inflammatory properties, including 3-O-Feruloylquinic acid, 15-deoxy-Δ-12,14-PGJ2, and 4-Hydroxy-3-methoxycinnamaldehyde [96,97,98], while other metabolites found in our fermented coffee have been reported as essential nutrients for a wide range of critical functions in the human body, including adenosine for heart and brain function [99,100]. This study indicates coffee fermentation results in the production of a range of metabolites beneficial for health, in addition to enhancing the flavor and aroma of the finished product.

## 4. Conclusions

Exotic coffees have been associated with numerous desirable traits (such as floral and fruity attributes, with decreased bitterness and acidity) and because the aroma, flavor, and metabolite profiles of coffee are easily impacted by the fermentation process during coffee processing, suitable starter cultures that consistently generate these attributes are valuable. Aroma is a key attribute that defines the quality, as well as the level of consumer acceptance, for coffee products. Yeast and bacterial fermentation of sugar-containing coffee extracts has been employed for the flavor development in ready-to-serve coffee beverages, but the role of fungi in fermentation and flavor development still needs elucidation. The extracellular enzymes and organic acids produced from fungi, yeast, and lactic acid and their metabolic activities during fermentation could potentially lead to the hydrolysis of macromolecules such as carbohydrates, proteins, and polyphenols to generate important aroma precursors, such as reducing sugars, amino acids, and chlorogenic acids. Secondary metabolites produced during the course of fermentation could also directly or indirectly affect coffee aroma.

The influence of numerous natural variables, including diverse microflora and processes, during coffee fermentation creates varieties of aroma and metabolites in the finished beverage. This work revealed the diverse metagenome of microorganisms in the wet process of coffee fermentation and their potential impact on flavor, aroma, and production of metabolites. From these data, we can correlate the relationship between the microbes and the biochemical compounds. A total of 64 genera of bacteria and 59 genera of yeast/fungi were found, with more diversity present in the slurry than in the cherries. Group of microbes, mainly yeast and lactic acid bacteria, that predominated in the process were correlated with preferable flavor and aroma compounds including linalyl formate, linalool, cis- isoeugenol, trans-geraniol, and (-)-isopulegol. From our study, *Lactobacillus* sp., *Lactococcus* sp., *Chryseobacterium* sp., *Enterobacter* sp., *Gluconobacter* sp., *Leuconostoc* sp., *Yokenella* sp., *Pichia* sp., *Torulaspora* sp., *Hanseniaspora* sp., *Alternaria* sp., and *Candida* sp. were predominant when the preferable flavor and aroma were generated. Of the variety of metabolites detected, some were found to be active compounds which could play a role in health. Traditionally, the coffee fermentation process relies on natural microflora present on coffee cherries and the environment, which can lead to inconsistency and uncontrollability of the coffee fermentation process. The production of a premium quality of coffee, based on superior properties of aroma, flavor, and metabolite profiles produced during fermentation could be developed based on the selection of a starter culture using the information obtained from this study.

## Figures and Tables

**Table 1:** Flavor and aroma compounds of the samples at different time points of fermentation analyzed by headspace stir bar sorptive extraction (HSSE) and gas chromatograph–mass spectrometer triple quadrupole (GCTQ).

Day	Compound	Chemical Formula	Flavor/Aroma	References
**Day 0 (Cherry)**	2-Oxohexanedioic acid	C_6_H_8_O_5_		
	2Z,6E-Farnesol	C_15_H_26_O		
	Propanoic acid, 2-methyl-, 2,2-dimethyl-1-(2-hydroxy-1-methylethyl) propyl ester (CAS)	C_12_H_24_O_3_		
	N-((2RS,3RS)-3-amino-2-butanyl) aniline	C_10_H_16_N_2_		
	1,3-Dimethyl-5,5-bis-(3-methyl-2-methylenebut-3-enyl) pyrimidine-2,4,6-trione	C_18_H_24_N_2_O_3_		
	6,6-Dimethyl-2-(1′-methyl-2′-carbomethoxy ethylbicyclo (3.1.1) hept-2-ene	C_14_H_22_O_2_		
	cis,exo-5n-Butyl-1,2,3,4,4a,8b-hexahydro-1,4-methanobiphenylene	C_17_H_22_		
	cis,exo-5n-Butyl-1,2,3,4,4a,8b-hexahydro-1,4-methanobiphenylene	C_17_H_22_		
**Day 0 (Slurry)**	Camphor (CAS)	C_10_H_16_O	Camphoreous	[34]
	linalyl formate	C_11_H_18_O_2_	Citrus, herbal, bergamot Lavender Soapy Fatty, green, woody	[35]
	(E,E)-3,7-Dimethyl-1-(methoxymethoxy)-1,6-octadien-3-ol	C_12_H_22_O_3_		
	Ascaridole	C_10_H_16_O_2_		
	Propanoic acid, 2-methyl-, 2,2-dimethyl-1-(2-hydroxy-1-methylethyl) propyl ester (CAS)	C_12_H_24_O_3_		
	1,3-Dimethyl-5,5-bis-(3-methyl-2-methylenebut-3-enyl) pyrimidine-2,4,6-trione	C_18_H_24_N_2_O_3_		
	Caffeine (CAS)	C_8_H_10_N_4_O_2_	Odorless	[36]
**Day 2 (Cherry)**	Linalool	C_10_H_18_O	Citrus, floral, sweet bois de rose Woody Green Blueberry	[37]
	(-)-Isopulegol	C_10_H_18_O	Minty cooling, medicinal Woody	[38] [39]
	2Z,6E-Farnesol	C_15_H_26_O		
	Cis-isoeugenol	C_10_H_12_O_2_	Sweet spicy, carnation Phenolic Floral	[40]
	1,3-Dimethyl-5,5-bis-(3-methyl-2-methylenebut-3-enyl) pyrimidine-2,4,6-trione	C_18_H_24_N_2_O_3_		
	Caffeine (CAS)	C_8_H_10_N_4_O_2_	Odorless	[36]
**Day 2 (Slurry)**	Linalool	C_10_H_18_O	Citrus Floral Sweet bois de rose Woody Green, blueberry	[37]
	Camphor (CAS)	C_10_H_16_O	Camphoreous	[34]
	Linalyl formate	C_11_H_18_O_2_	Citrus, herbal, bergamot Lavender, soapy, fatty, green Woody	[35]
	2,4-dimethyl-3,6-dihydro-2H-pyran	C_7_H_12_O		
	3-Hydroxy-2,2-dimethylhexyl ester of butanoic acid	C_12_H_24_O_3_		
	trans-Caryophyllene	C_15_H_24_		
	1,3-Dimethyl-5,5-bis-(3-methyl-2-methylenebut-3-enyl) pyrimidine-2,4,6-trione	C_18_H_24_N_2_O_3_		
	Caffeine (CAS)	C_8_H_10_N_4_O_2_	Odorless	[36]
**Day 4 (Cherry)**	Camphor (CAS)	C10H16O	Camphoreous	[34]
	trans-Geraniol	C_10_H_18_O	Sweet floral, fruity Rose Waxy Citrus	[41]
	Propanoic acid, 2-methyl-, 2,2-dimethyl-1-(2-hydroxy-1-methylethyl) propyl ester (CAS)	C_12_H_24_O_3_		
	1-Octadecanol (CAS)	C_18_H_38_O	Bland	[42]
	Caffeine (CAS)	C_8_H_10_N_4_O_2_	Odorless	[36]
**Day 8 (Cherry)**	(-)-Isopulegol	C_10_H_18_O	Minty cooling, medicinal Woody	[38] [39]
	1-cyclopropyl-4-methoxybenzene	C_10_H_12_O		
	trans-Geraniol	C_10_H_18_O	Sweet floral, fruity Rose Waxy Citrus	[41]
	Propanoic acid, 2-methyl-, 2,2-dimethyl-1-(2-hydroxy-1-methylethyl) propyl ester (CAS)	C_12_H_24_O_3_		
	1,3-Dimethyl-5,5-bis-(3-methyl-2-methylenebut-3-enyl) pyrimidine-2,4,6-trione	C_18_H_24_N_2_O_3_		
	Caffeine (CAS)	C_8_H_10_N_4_O_2_	Odorless	[36]

## Data Availability

The original contributions presented in the study are included in the article, further inquiries can be directed to the corresponding author.
